# Regulatory T-Cell-Mediated Suppression of Conventional T-Cells and Dendritic Cells by Different cAMP Intracellular Pathways

**DOI:** 10.3389/fimmu.2016.00216

**Published:** 2016-06-02

**Authors:** Cesar M. Rueda, Courtney M. Jackson, Claire A. Chougnet

**Affiliations:** ^1^Division of Immunobiology, Department of Pediatrics, Cincinnati Children’s Hospital Research Foundation, University of Cincinnati College of Medicine, Cincinnati, OH, USA

**Keywords:** cyclic adenosine monophosphate, T regulatory cells, suppression, dendritic cells, T cells

## Abstract

Regulatory T-cells (Tregs) mediate their suppressive action by acting directly on conventional T-cells (Tcons) or dendritic cells (DCs). One mechanism of Treg suppression is the increase of cyclic adenosine 3′,5′-monophosphate (cAMP) levels in target cells. Tregs utilize cAMP to control Tcon responses, such as proliferation and cytokine production. Tregs also exert their suppression on DCs, diminishing DC immunogenicity by downmodulating the expression of costimulatory molecules and actin polymerization at the immunological synapse. The Treg-mediated usage of cAMP occurs through two major mechanisms. The first involves the Treg-mediated influx of cAMP in target cells through gap junctions. The second is the conversion of adenosine triphosphate into adenosine by the ectonucleases CD39 and CD73 present on the surface of Tregs. Adenosine then binds to receptors on the surface of target cells, leading to increased intracellular cAMP levels in these targets. Downstream, cAMP can activate the canonical protein kinase A (PKA) pathway and the exchange protein activated by cyclic AMP (EPAC) non-canonical pathway. In this review, we discuss the most recent findings related to cAMP activation of PKA and EPAC, which are implicated in Treg homeostasis as well as the functional alterations induced by cAMP in cellular targets of Treg suppression.

## Introduction

Regulatory T-cells (Tregs), first described by Sakaguchi et al. in 1995 (1), are essential to maintain immune homeostasis and protection against autoimmunity. This CD4^+^ T-cell subset highly expresses IL-2 receptor alpha chain (CD25) and Forkhead box P3 (FOXP3), the central transcription factor for Treg development and function. Defects in the FOXP3 gene in both mice and humans lead to a fatal lymphoproliferative and autoimmune disease (2, 3).

Regulatory T-cells control immune activation by acting directly on conventional CD4^+^ and CD8^+^ conventional T cells (Tcons) and antigen-presenting cells, such as dendritic cells (DCs). Tregs preferentially localize to DC aggregates, subsequently preventing Tcon activation *in vivo* and *in vitro* (4, 5), suggesting DCs are the primary targets of Treg suppression (6, 7). Cyclic adenosine 3′,5′-monophosphate (cAMP) was recognized in 2007 as being essential to Treg suppression (8). cAMP is a common intracellular second messenger found in various cell types, which was discovered in the year 1957 (9). It is generated after the initial binding of hormones, neurotransmitters, and other ligands to cell-surface receptors (10). cAMP activates the canonical protein kinase A (PKA) pathway and the exchange protein activated by cyclic AMP (EPAC) non-canonical pathway (11, 12). In this review, we will discuss how cAMP regulates Tcon and DC function, as well as describing downstream PKA and EPAC intracellular pathways within Tregs, Tcons, and DCs.

## Elevated cAMP Concentration in Tregs is Determined by Adenylyl Cyclase and Phosphodiesterase Expression

Intracellular cAMP levels are regulated by adenylyl cyclases (ACs) that catalyze the formation of cAMP and phosphodiesterases (PDEs), which hydrolyze cAMP to 5′-AMP. Overall, there are 11 PDEs and 10 AC families. ACs 3, 6, 7, and 9 are expressed in murine T cells (13, 14). PDEs 3, 4, 7, and 8 are expressed in human T-cells, with PDE4 being the most abundant (15–17). Importantly, the differential expression and activation of ACs and PDEs in Tregs and Tcons explain the high level of intracellular cAMP in murine and human Tregs compared to Tcons (8, 18, 19).

**Figure 1:**
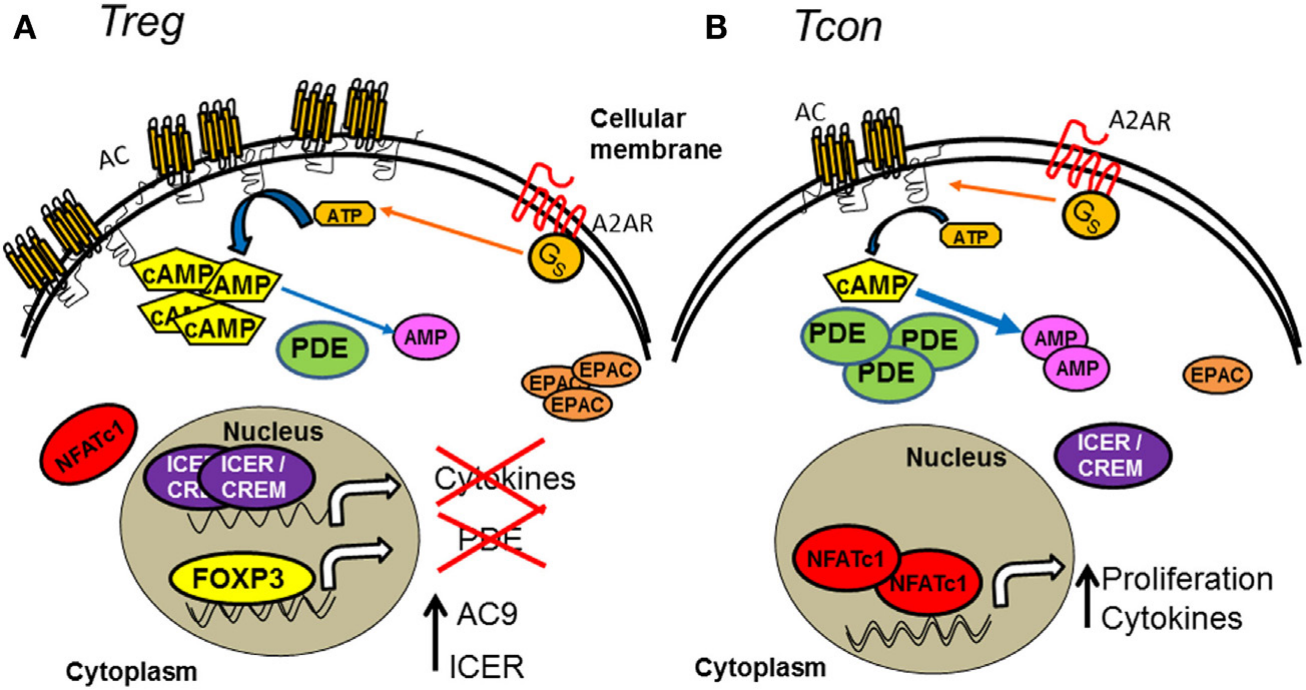
**Comparison of cAMP metabolism and intracellular signaling pathway in Treg and Tcon subsets**. **(A)** Tregs contain a high concentration of cAMP compared to Tcons as a consequence of their high cAMP anabolism. Tregs express mainly AC compared to Tcons, and AC catalyzes the conversion of ATP into cyclic adenosine monophosphate (cAMP). In addition, G protein-coupled receptors, such as A_2A_, are able to activate AC. In contrast to Tcons, Tregs exhibit low cAMP catabolism due to a low expression of PDEs, which decompose cAMP into AMP. The presence of FOXP3 in Tregs, but not in Tcons, suppresses PDE transcription, while it favors ICER and AC expression. The localization and expression of molecules, such as ICER/CREM (high expression and nuclear), NFAT (low expression and cytoplasmic) and EPAC (high expression), are associated with the maintenance of Treg phenotype and function. **(B)** Tcons contain low levels of cAMP due to their reduced AC but high PDE expression. In contrast to Treg, NFAT is active in the nucleus of Tcons. The low and cytoplasmic expression of ICER/CREM and EPAC are also associated with cell cycle progression and active cytokine secretion in Tcons.

Similar to its expression in murine Tregs, AC7 is expressed in resting and activated human Tregs (20). Activation of AC7 downstream of IL-2 signaling plays an important role in promoting high cAMP levels in resting Tregs (18). However, since CD25 expression is upregulated in Tcons following activation, preferential IL-2-mediated AC7 activation is not sufficient to explain the increased cAMP levels present in activated Tregs compared to activated Tcons. Elevated expression of AC9 has also been shown to be important for cAMP accumulation in murine Tregs (13) (Figure 1A), which is regulated in part by microRNA miR-142-3p targeting of AC9 mRNA expression. Although FOXP3 downregulates miR-142-3p to keep the AC9/cAMP pathway active in Tregs (13), miR-142-3p is elevated in other CD4^+^ subsets, keeping AC9 inactive and thus cAMP levels low. Additionally, an isoform of PDE (PDE3b) is one of the most FOXP3-repressed genes in murine Treg (21), resulting in low cAMP degradation and subsequent elevation of cAMP levels in Tregs (Figure 1A). Further demonstrating the involvement of FOXP3 in cAMP regulation, T cells programed to be Tregs, but that did not express functional FOXP3 protein due to a frame-shift mutation, had substantially lower intracellular cAMP levels than FOXP3-expressing Tregs (22). However, we recently reported that neonatal human Tregs have lower expression of FOXP3, but higher intracellular cAMP levels compared to adult Tregs, suggesting that cAMP levels may also be regulated in a FOXP3-independent manner (23). Several mechanisms may explain this profile exhibited by human neonatal Tregs, and neonatal plasma contains high adenosine concentrations due to a low degradation rate (24, 25). In addition, the adenosine receptors in neonatal mononuclear cells seem to be more sensitive than those in adults, leading to higher intracellular cAMP (24, 25).

## Tregs Increase cAMP Levels in Target Cells through cAMP Influx and Adenosine Production

The ability of Tregs to generate and accumulate high levels of cAMP gives them the capacity to transfer it through gap junctions (GJ) into target cells (8, 19, 26, 27). These channels allow intercellular exchange of ions, metabolites, and other molecules between adjacent cells (28). GJ are formed by two opposing hemichannels from each cell, called connexons, which are made of six proteins called connexins (Cx) (28). Tregs and Tcons both express Cx31.1, Cx32, Cx43, Cx45, and Cx46, and their expression increases after activation (8).

An additional mechanism to increase cAMP in the target cell involves the ecto-5′-nucleotidases CD39 and CD73 expressed on the surface of Tregs, which cleave extracellular adenosine triphosphate (ATP) into adenosine (29, 30). The binding of adenosine to its G protein-coupled receptors (GPCRs) on target cells leads to the stimulatory G protein alpha subunit (G_sα_) directly activating ACs and generating cAMP (31–33).

Extracellular ATP, which is a hallmark of inflammation (34, 35), is first degraded to AMP by CD39, which CD73 converts into adenosine (36). CD39 and CD73 are coexpressed on the surface of murine Tregs (29, 37). In contrast to murine Tregs, very few (<5%) human CD39^+^ Tregs appear to express CD73 on their surface (38–41). However, there is high intracellular expression of CD73 in human Tregs, which seems to be readily shed from their surface, potentially explaining the low surface expression of CD73 on human Tregs (39). Furthermore, human Tregs produce exosomes that carry CD39 and CD73 and are able to hydrolyze ATP (42–44).

Adenosine binds to several receptors (A_1_, A_2A_, A_2B_, and A_3_) that are expressed on various cells, including T cells and APCs. High affinity A_2A_ appears to be the main receptor involved in mediating adenosine-dependent Treg suppressive function (39). Binding of adenosine to A_2A_ on target cells, such as T cells and APCs, activates AC, leading to cAMP accumulation (33, 45). The A_2A_ receptor is also expressed by resting and activated Tregs (30) and treatment for Tregs by adenosine analogs increased their cAMP levels (46) (Figure 1A). A_2A_ stimulation not only expanded FOXP3^+^ Treg cells but also increased their suppressive function (46, 47). The fact that Tregs produce and respond to adenosine thus suggests that adenosine might act as an autocrine factor to optimize Treg anti-inflammatory function, as proposed by Ernst et al. (48).

## The Role of cAMP in Treg Control of Tcon Proliferation and Cytokine Production

Pharmacological inhibition of PDE3 and PDE4 increases cAMP levels in Tregs and leads to their enhanced suppression of Tcons, both *in vivo* and *in vitro* (49, 50). Conversely, treatment for human Tregs with interferon-α before activation decreased intracellular cAMP through PDE4 activation, which led to a loss of Treg suppression (51). Tregs were shown to inhibit *in vitro* activation of Tcons by using the mechanisms, as described previously, e.g., by transferring cAMP through GJ (8, 30) and *via* CD39-mediated generation of adenosine. We have shown that Tregs limit *in vitro* HIV infection in Tcons using these same two mechanisms (40).

The relevance of CD39 expression as a mechanism of suppression was also shown in Tregs from CD39-null mice, which failed to suppress CD4^+^CD25^−^ cell proliferation (30). In addition, in a murine melanoma model, the adenosine generated by the increased frequency of CD39^+^ Tregs was associated with the suppression of T-cell effector functions (52). Similarly, human CD39^+^ Tregs suppressed IL-2 and IL-17 expression and proliferation of activated Tcons more efficiently than their CD39^−^ counterparts (53–55).

## The Role of cAMP in Treg Control of DC Function

Regulatory T-cells also use cAMP to downregulate the expression of several costimulatory molecules on DCs, such as CD40, CD80, CD86, and CD83 (23, 26, 27, 29, 56–58), while upregulating the expression of several inhibitory molecules (B7-H, B7-H3, and B7-DC) (26, 29, 56). In contrast, cAMP did not modify cytokine production by DCs (26). Again, both mechanisms (cAMP influx and adenosine) appear to be active in this suppression (29, 56, 58).

## Tregs Control DC–Tcon Interactions through cAMP-Dependent Mechanisms

The duration of contact between Tcons and antigen-loaded DCs are shortened in the presence of Tregs (7), indicating an early effect of Tregs on the induction of immune responses. Additionally, Tregs are more mobile than Tcons *in vitro* and out-compete the latter in aggregating around DCs (5). Tregs were also found to form long-lasting conjugates with islet antigen-bearing DCs, which lost the capacity to effectively present antigens (4). Confirming the idea that DCs are the primary targets of Treg suppression, the amount of cAMP transferred from Tregs to DCs was significantly higher than that transferred from Tregs to Tcons in *in vitro* cocultures with DC–Tcon–Treg (56). In concordance with this, we have recently shown that cAMP, together with CTLA-4 and TGF-β, are essential for adult Tregs to suppress the formation of DC–Tcon aggregates (23). We have also shown that the influx of cAMP by adult Tregs suppresses actin polymerization at the interface of DCs and Tcons (23, 57). Importantly, due to the role of the immunological synapse in the transmission of HIV particles from DCs to Tcons, we showed that Tregs could blunt this transmission by cAMP-dependent mechanisms (57).

Interestingly, Ring et al. showed that Tregs specifically directed the migration of DCs toward them (58) and that adenosine played a major role in this phenomenon because CD39^−^ Tregs were unable to attract DCs (58). These data are consistent with the fact that cAMP could stimulate DC chemotaxis by increasing the expression of the lymph node-homing chemokines CCL19 and CCL21 (59). Taken together, these data suggest that Tregs use cAMP at multiple levels to prevent Tcon activation by DCs, they not only keep the DCs in an immature state but they also attract them away from Tcons.

## Treg-Mediated Suppression of Tcons and DCs by Different Intracellular Pathways Downstream of cAMP

### PKA and EPAC Intracellular Signaling Pathways

Once inside the cell, cAMP triggers various downstream pathways, mainly the canonical PKA and the non-canonical EPAC pathway. PKA contains an evolutionarily conserved cAMP-binding domain (CBD) that acts as a sensor of intracellular cAMP levels (11). cAMP binding to CBD on the PKA regulatory subunit induces its activation and releases the catalytic subunit. Downstream of PKA dissociation, several signaling pathways are regulated (activated or inactivated) by the catalytic subunit of PKA through phosphorylation. For example, cAMP response element binding protein (CREB) is phosphorylated at serine133 by PKA, leading to the complex formation of CREB with CSK (CBP) (60, 61). This complex binds to cAMP responsive elements in the promoter regions of genes such as cAMP response element modulator (CREM). CREM regulates multiple transcriptional activators or inhibitors in T cells. Increased cellular cAMP levels enhance the expression of an isoform of CREM, named the inducible cAMP early repressor (ICER). cAMP promotes ICER translocation from the cytoplasm to the nucleus (62). This translocation is crucial for ICER action because ICER binds activator protein-1 (AP-1) and nuclear factor of activated T-cells (NFAT) in the nucleus. These nuclear interactions between ICER and NFAT/AP-1 mediate ICER transcriptional regulation of many genes involved either in cell cycle control or cytokine secretion (63, 64).

Cyclic adenosine 3′,5′-monophosphate can also activate the non-canonical EPAC pathway. EPAC proteins (EPAC1 and EPAC2) contain a CBD that is homologous to the one contained within PKA. cAMP binding to EPAC proteins activates the Ras superfamily small GTPases RAP-1 and RAP-2 by promoting the exchange of GDP for GTP (11). Depending on the cell type, EPAC and PKA may act independently or synergistically or oppose each other in regulating specific cellular functions (11).

### Activation of cAMP–PKA and cAMP–EPAC Control the Phenotype and Suppressive Activity of Tregs

Several authors have shown that the cAMP–PKA pathway in Tregs differs from that in Tcons. Although the expression of PKA is similar in Tregs and Tcons (12), other molecules, such as ICER/CREM, are markedly more expressed in Tregs than in Tcons (65). In addition, ICER/CREM is mainly localized in the nucleus in Tregs (Figure 1A) (65). This particular phenotype in Tregs may be explained by the presence of FOXP3, as forced expression of FOXP3 in murine Tcons induced constitutive expression of ICER/CREM (66). cAMP signaling and the presence of ICER in the nucleus seems to be important to blunt Treg cytokine production (19). However, deletion of ICER did not alter the number or function of murine Tregs (12). In contrast to ICER/CREM, NFATc1 is primarily localized in the cytoplasm of Tregs (65, 67, 68) (Figure 1A). Decreasing cAMP increased induction and nuclear translocation of NFATc1 in human Tregs, leading to increased Treg proliferation and blunted suppression (19). cAMP/PKA signaling in Tregs also modulates the expression of other functionally important molecules, such as CTLA-4 (69). Furthermore, PKA/CREB activation promotes the TGFβ-mediated generation of Tregs from naive Tcons and contributes to the maintenance of FOXP3 expression in these induced Tregs both *in vitro* and *in vivo* (69–71).

Murine Tregs express 10-fold more EPAC1 than naive and activated Tcons (12) (Figure 1A). In contrast to ICER, EPAC1 is critical for Treg-mediated suppression as genetic deletion of EPAC1 attenuated their suppression of Tcons (72). Furthermore, RAP-1 seems to be more activated in human Tregs compared to Tcons (73). Transgenic mice with a constitutively active RAP-1 have increased Treg frequency, and their Tregs are more suppressive (74, 75). These data suggest a feedback cycle in Tregs (particularly in peripheral Tregs), whereby sustained and elevated levels of cAMP and FOXP3 expression intensify each other, helping Tregs maintain their phenotype and functionality.

### Tregs Induce PKA and EPAC Activation in Tcons

**Figure 2:**
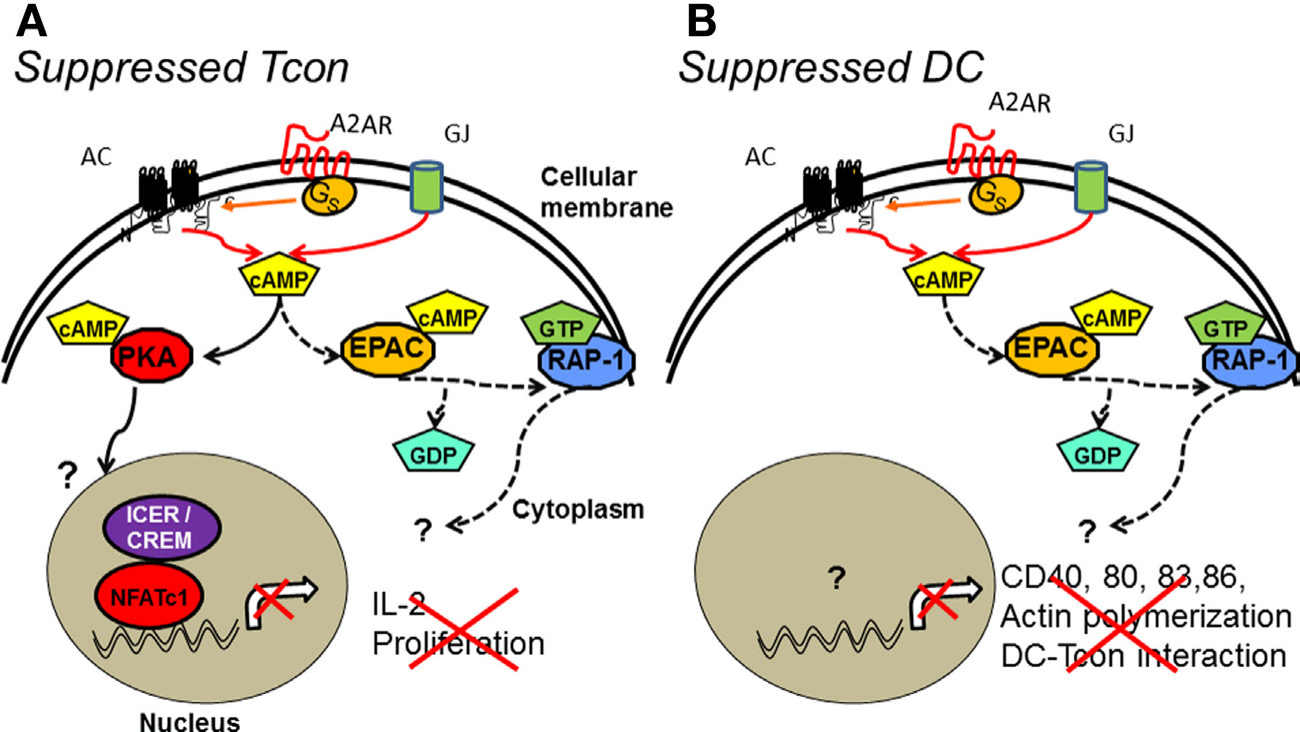
**cAMP intracellular signaling pathways in target cells of Treg suppression**. Activation of AC induced by adenosine interaction with its receptor (A_2A_) and/or influx of cAMP through gap junctions (GJ) increases cAMP concentration in Treg target cells. **(A)** cAMP-mediated suppression by Treg decreases effector responses (cytokine production and proliferation) in Tcon by PKA and EPAC. PKA activation in Tcon results in the translocation of ICER/CREM into the nucleus inhibiting NFATc1-driven transcription. Downstream signaling in the EPAC pathway includes the activation of the small GTPase RAP-1, promoting the exchange of GDP for GTP. **(B)** In DCs, the suppression of costimulatory molecules expression, actin polymerization, and DC–Tcon interaction downstream of cAMP seems to be due to EPAC activation. Intermediate and final molecules downstream of PKA and RAP-1, respectively, are still unknown.

Interestingly, the intracellular signaling pathways downstream of cAMP appear to differ between target cells. Tregs redundantly induce the activation of PKA and EPAC in Tcons. The role of PKA in blunting Tcon proliferation was demonstrated through the use of cAMP analogs that bind PKA (76), long before Tregs were discovered to use this pathway to blunt Tcon activation. Downstream of PKA, ICER/CREM is normally localized in the cytoplasm of Tcons (Figure 1B). These molecules translocate and accumulate within the nucleus of Tcons in presence of Tregs, blunting IL-2 synthesis (65) (Figure 2). However, ICER^−/−^ Tcons were still significantly suppressed by wild-type Tregs, suggesting that EPAC may also contribute to Treg suppression (12). Confirming this hypothesis, EPAC1^−/−^ Tcons were poorly suppressed by Tregs (72). Taken together, these data suggest that Tregs induce both PKA and EPAC activation in Tcons, and these mechanisms could work cooperatively to suppress Tcon activation.

### Tregs Induce EPAC Activation in DCs

Exchange protein activated by cyclic AMP proteins regulate many mechanisms in APCs, such as integrin-dependent cell adhesion, polarization, chemotaxis, and phagocytosis (77, 78). Therefore, not surprisingly, triggering the cAMP–EPAC1 axis through Treg-derived adenosine activated RAP-1–GTP and increased DC migration (58). Tregs were also able to induce a re-localization of RAP-1 to the membrane of DCs, from its cytoplasmic localization when DCs are not in contact with Tregs (58) (Figure 2B). By contrast, Tregs do not prevent CREB activation in DCs, suggesting that Treg-mediated DC suppression is not PKA dependent (58). Similarly, cAMP does not inhibit the PKA/CREB-dependent production of inflammatory chemokines CCL3 and CCL4 by DCs (79). In addition, PKA activation by cAMP analogs induces maturation of human DCs, as evidenced by the increased surface expression of MHC class II, costimulatory molecules, and CD83 by treated DCs (80). These studies thus suggest that increased cAMP levels in DCs due to contact with Tregs is suppressive; however, PKA and EPAC appear to mediate opposing effects in DC.

The mechanisms that explain these cell-specific differential effects are still unclear. Interestingly, although pharmacological cAMP analogs that directly bind PKA are active in DCs, EPAC activity in DC appears to suppress PKA activation (80), which could thus explain why cAMP is globally suppressive in DCs. Second, the cellular effect of cAMP may also vary depending on the relative cellular abundance/distribution of EPAC and PKA and/or the amount of cAMP injected by Tregs in the target cells (56, 81). Finally, EPAC and PKA have opposite regulatory effects on downstream targets such as PKB (81). Activation of EPAC leads to a phosphatidylinositol 3-kinase-dependent PKB activation, whereas stimulation of PKA inhibits PKB activity (81). Future experiments are required to evaluate what controls cAMP downstream pathways in a cell-specific context.

## Conclusion

It is now accepted that the cAMP-dependent intracellular signaling induced by Tregs in target cells is much more complex than initially assumed, and the classic PKA pathway is only part of the story. Although both human and murine Tregs mediate suppression indistinctly by cAMP influx and/or the CD39/adenosine pathway, the downstream pathways differ in Tcons and DCs. On the one hand, Treg suppression of Tcon cytokine production and proliferation requires the integration of EPAC and PKA in a cooperative manner. On the other hand, the suppression of DC function seems to be mainly mediated by EPAC, with a paradoxical opposite effect of PKA. Although our knowledge of the EPAC pathway in DCs has greatly progressed in the past years, much remains to be discovered. In particular, we still lack a full understanding of the physiological role of EPAC and RAP isoforms, of the mechanisms of PKA inactivation, and of the effector molecules downstream of RAP-1. A cautious dissection of the individual role and comparative contribution of EPAC and PKA within the overall cAMP signaling in various models will continue to be an important goal for upcoming investigations. This could lead to the development of targeted approaches fine-tuning Treg suppression for therapeutic applications.

## Author Contributions

CR and CJ wrote the manuscript, and CC discussed and edited the manuscript.

## Conflict of Interest Statement

The authors declare that the research was conducted in the absence of any commercial or financial relationships that could be construed as a potential conflict of interest.
